# Changes in abdominal subcutaneous adipose tissue thickness associate with disease and anthropometric factors

**DOI:** 10.1038/s41366-025-01829-y

**Published:** 2025-07-09

**Authors:** Marjola Thanaj, Nicolas Basty, Madeleine Cule, Elena P. Sorokin, Brandon Whitcher, Ramprakash Srinivasan, Jimmy D. Bell, E. Louise Thomas

**Affiliations:** 1 Research Centre for Optimal Health, School of Life Sciences, University of Westminster, London, UK; 2 Calico Life Sciences LLC, South San Francisco, CA USA

**Keywords:** Risk factors, Cardiovascular diseases

## Abstract

**Background:**

Three-dimensional (3D) mesh-derived phenotypes enable detailed characterisation of organ morphology and regional variation through statistical parametric maps (SPMs) and statistical shape analysis (SSA). While these techniques have been widely used for organ studies, their application to abdominal subcutaneous adipose tissue (ASAT) has been limited. This study investigates the associations between ASAT thickness, anthropometric traits, and clinical conditions, including type 2 diabetes (T2D) and hypertension.

**Methods:**

We analysed ASAT using MRI data from 44,515 participants in the UK Biobank who underwent baseline imaging, with a subset of 3088 participants receiving a follow-up scan approximately 2 years later. ASAT thickness was quantified using 3D surface meshes. Regional associations with anthropometric and clinical variables were examined using SPMs. Additionally, principal components of ASAT thickness, derived via SSA, were analysed for their association with future cardiovascular disease (CVD) risk.

**Results:**

ASAT thickness was significantly associated with age, alcohol consumption, visceral fat, total muscle mass, and various health-related traits. Longitudinal analysis revealed significant changes in ASAT thickness over a 2.5-year period in both sexes, independent of disease status at baseline. Notably, regional variations in hip ASAT thickness were associated with incident CVD in women (hazard ratio [HR]: 0.90, 95% CI: 0.84–0.97, *p* = 0.023) and with hypertension in both women (HR: 1.10, 95% CI: 1.03–1.21, *p* = 0.045) and men (HR: 0.88, 95% CI: 0.82–0.96, *p* = 0.014).

**Conclusions:**

3D quantification and morphometric analysis of ASAT offer novel insights into the associations between abdominal fat distribution, lifestyle factors, and chronic disease risk. These techniques hold promise for enhancing our understanding of fat-related disease mechanisms in population-level studies.

## Introduction

Obesity is commonly recognised as a global health burden [1]. The distribution of adipose tissue (AT) in the human body is uniquely important for understanding human metabolism [2]. While visceral adipose tissue (VAT) is known for its highly metabolic activity and strongly associated with metabolic disorders [3], the contribution from abdominal subcutaneous adipose tissue (ASAT) is generally considered more benign or even protective in terms of contribution to metabolic risk [4, 5]. However, emerging evidence suggests that higher levels of pro-inflammatory cytokines secreted by ASAT, particularly in higher levels of obesity, suggest it has a more detrimental role [6, 7]. Consequently, a more in-depth understanding of ASAT is vital for effectively phenotyping individuals at an elevated risk for metabolic disease.

Magnetic resonance imaging (MRI) is widely adopted for the precise quantification of different AT depots [8], facilitated by recent advances in automated analysis that now enable routine measurement of ASAT (and VAT) volumes at the population level [9]. However, while these automated techniques provide accurate volumetric measurements, they fall short of capturing detailed information on morphological, functional, and regional variations in response to specific health conditions.

Three-dimensional (3D) mesh-derived phenotypes provide valuable insights into morphological and regional organ variations through statistical parametric maps (SPMs), enabling the detection of differences between healthy and diseased states. Similarly, statistical shape analysis (SSA) can transform spatially correlated data into a smaller set of principal components to characterise organ shape variations across a population. These morphometric analyses offer a non-invasive means of modelling the human body and have been extensively used across multiple organs [10–14]. However, they have been less frequently applied to the study of AT.

In this study, we quantify three-dimensional (3D) ASAT mesh-derived phenotypes from MRI scans. Our first objective was to measure ASAT thickness at all surface vertices and identify morphological variations using statistical parametric maps (SPMs) in relation to anthropometric traits and clinical conditions, including type 2 diabetes (T2D) and hypertension. Second, we examined longitudinal changes in ASAT thickness over a 2.5-year period to assess whether these changes are influenced by clinical factors. Third, we applied dimensionality reduction techniques to extract shape features from the 3D ASAT meshes and evaluated their association with future risk of disease outcomes.

## Methods

### Data

The UK Biobank is a population-based study in which approximately 60,000 participants underwent abdominal MRI scans, between 2014 and 2020, with over 3000 having undergone a repeat imaging visit after approximately two and a half years. Full details regarding the UK Biobank abdominal MRI acquisition protocol and the repeat scanning have previously been reported [15, 16]. Briefly, the data included here focused on the neck-to-knee chemical shift imaging (also known as Dixon MRI) acquisitions involving six overlapping series. The Dixon MRI data were processed and segmented using automated methods as previously described [9]. Further information regarding the ethical approval can be found in the Data section of the Supplementary Material.

### Phenotype and disease definitions

Phenotypic data were obtained through screening questionnaires, interviews, body composition measurements, lifestyle assessments, hospital records, and primary care data (see Phenotype Definition in the Supplementary Material). Diseases were selected based on their established or hypothesised links to adipose tissue health and their availability within the UK Biobank dataset. We focused specifically on type 2 diabetes (T2D), cardiovascular disease (CVD), and hypertension, given their known associations with alterations in adipose tissue distribution and function^9^. Full disease definitions are provided in the Disease Definitions section and Supplementary Table S1.

### Image analysis, registration and mesh construction

A critical step in our analysis involved calculating the regional ASAT thickness at each vertex of the 3D surface mesh. Preprocessing was applied to ensure that tissue in the arms below the shoulders were removed from all the scans and only tissue associated with ASAT and the body cavity segmentations were included for any subsequent analyses. In brief, we truncated the arms at the armpits from both ASAT segmentations based on specified anatomical landmarks (including landmarks from multiple bone joints in the body) and masked the regions of interest by comparing the absolute difference between the truncated masked volume and the original volume. This meticulous preprocessing allowed us to obtain robust ASAT segmentations with no contamination from other anatomical areas.

The process for organ template construction has been previously detailed [13, 17]. Here, we constructed a template using the ASAT segmentations from a cohort of 20 participants, which serves solely as a referencing space. We then constructed 3D surface meshes from the template image and all participants’ segmentations using the marching cubes algorithm and smoothed using a Laplacian filter [18].

The registration process we employed has been previously outlined [13]. In brief, we used a multi-step registration process, including rigid, affine, and non-rigid registration, to align participant surfaces with a template. The template mesh was then propagated to each participant’s mesh, ensuring a consistent number of vertices (~90,000) and anatomical accuracy across all participants. These registration steps were performed using the Image Registration Toolkit (IRTK) (https://biomedia.doc.ic.ac.uk/software/irtk).

To determine the regional ASAT thickness at each vertex in the 3D model, the Euclidean distance between the outer layer of the ASAT segmentation and the body cavity was measured, for each subject (Supplementary Fig. S1).

### Quality control

This study included MRI data from 47,603 participants at their initial imaging visit, and 3088 participants who also underwent a follow-up scan approximately 2.5 years later. From the initial cohort, 5979 datasets were excluded due to missing anthropometric or lifestyle data. An additional 648 participants were removed due to preprocessing errors during landmark identification (e.g., potential participant misalignment). Further quality control was performed through visual inspection of outliers, defined as exceeding the 99.9th percentile of ASAT thickness. A threshold of 154 mm was set, resulting in the exclusion of 97 additional datasets. The final baseline sample included 37,888 participants. For the follow-up group, 702 datasets were excluded due to missing anthropometric variables, yielding a final longitudinal sample of 2386 participants. A detailed flow diagram of the quality control process is provided in Supplementary Fig. S2.

### Mass univariate regression analysis

Associations between ASAT thickness and anthropometric variables were estimated using a linear regression framework [19]. We applied threshold-free cluster enhancement (TFCE) [20] and permutation testing to assess the associations between ASAT thickness and anthropometric variables, and derive the *p* values associated with each regression coefficient following adjustment for relevant covariates with the correction to control the false discovery rate (FDR), as previously described [17] (see Mass Univariate Regression Analysis in the Supplementary Material and Supplementary Fig. S3).

To determine which factors were linked with ASAT thickness, we performed a sex-stratified analysis included adjustments for covariates like age, ethnicity, height, hand grip strength (HGS) in the dominant hand, Townsend deprivation index, alcohol intake frequency, smoking status, vigorous physical activity as measured in metabolic equivalent of task (MET) units, total muscle volume, VAT volume, lipodystrophy, defined from fat-to-muscle-ratio (FMR) > 1.2 (F)/ > 1.7 (M) [21] (see Phenotypes Definitions in Supplementary Material), and disease including T2D and hypertension (see Disease Definitions in Supplementary Material), excluding BMI and CVD due to correlation with ASAT and being included in the definition for hypertension, respectively. Total muscle volume was quantified within the neck-to-knee region, using our previously published automated pipeline from Dixon MRI scans [22]. Model summaries are reported as median regression coefficients ($\hat{\beta }$) along with the corresponding significance areas, representing the percentage of which the $\hat{\beta }$ is statistically significant (*p* < 0.05) across all vertices in the mesh. All continuous variables, including ASAT thickness, were standardised prior to being included in the regression models.

### Longitudinal mesh analysis

We analysed 2386 participants that had undergone a second imaging visit after 2.3 (0.3) years (median (interquartile range—IQR)). The Wilcoxon rank-sum test was used to compare means between imaging visits. To evaluate changes in ASAT thickness over time, a linear mixed-effects models were applied to ASAT thickness at each vertex of the 3D ASAT surface mesh, separately for men and women, using the *lme4* package [23]. Participant IDs were included as a random effect, while disease/conditions and other covariates (age, ethnicity, height, dominant HGS, Townsend deprivation index, alcohol intake, smoking, vigorous MET, total muscle, and VAT) were fixed effects. Data from both time points were included instead of performing analysis on the differences [24]. The models assessed the main effects of imaging visit and condition/disease, including their interaction term. *p* values for the regression coefficients in the linear mixed-effects models were computed using the *lmerTest* package [25], with FDR correction (threshold <0.05) for multiple comparisons. All continuous variables fixed effects were standardised.

### Survival analysis

Cox proportional hazards (CPH) models were used to assess the associations between the 3D mesh-derived phenotype and the incident risk of each disease outcome that occurred after the imaging visit. To reduce the dimensionality of the 3D mesh-derived phenotypes we computing the robust sparse principal component analysis (SPCA) (see Statistical Shape Analysis in Supplementary Material) on ASAT thickness, across male and female participants separately, within each disease cohort and extracted the principal component (PC) scores. We conducted CPH models separately for each disease outcome and gender. The first model (volume model) included age, ethnicity, height, dominant HGS, Townsend deprivation index, alcohol intake, smoking, vigorous MET, VAT, and ASAT volume. The second model (thickness model) replaced ASAT volume with the principal component (PC) scores of ASAT thickness. Total muscle was excluded due to correlation with PCs.

Disease outcomes and dates of the first occurrence of CVD, hypertension and T2D are defined in Supplementary Table S1. Time-to-event was censored at the first event for each outcome, death, or last recorded follow-up (31th of November 2022), with 4.6 (2.4) years (median (IQR)) follow-up period since the imaging visit. Participants with an event recorded prior to the imaging visit were excluded.

Model summaries are reported as hazard ratios with 95% confidence intervals (CIs) and FDR correction (threshold <0.05) was applied for multiple comparisons. Model comparisons used Akaike Information Criterion (AIC) and concordance index (c-index), where lower AIC indicated better model fit and higher c-index suggested better predictive accuracy. All continuous variables were standardised before analysis.

## Results

### Study population characteristics

Of the cohort of 37,888 participants, 96.9% were white, with an age range between 44 and 82 years, and a mean BMI 26.0 ± 4.7 kg/m^2^ for women and 27.0 ± 3.9 kg/m^2^ for men (Supplementary Table S2). Lipodystrophy was identified in 2050 women and 2252 men. Additionally, 692 women and 1376 men had T2D, while 5794 women and 8342 men had hypertension.

### Associations with anthropometric traits and disease

**Fig. 1:**
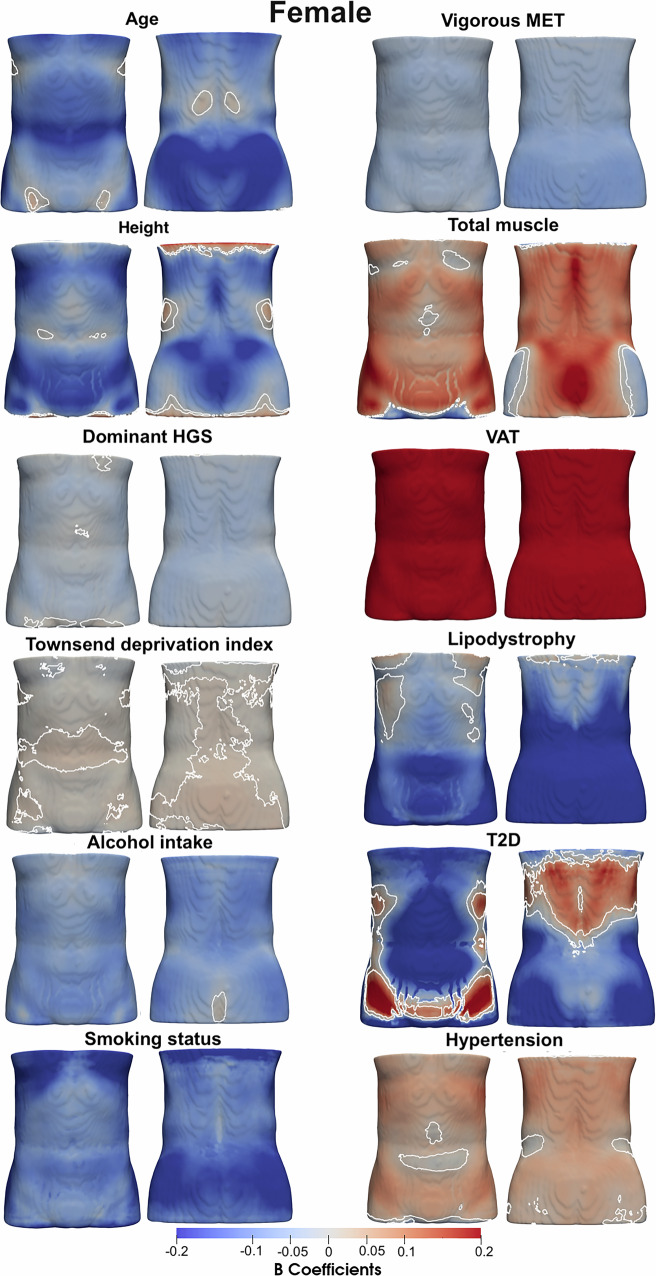
Three-dimensional statistical parametric maps (SPMs) of ASAT morphology, projections are anterior (left plots) and posterior views (right plots). The SPMs show the local strength of association for each covariate in the model with ASAT thickness for female participants (*N* = 19,418). White contour lines indicate the boundary between statistically significant regions (*p* < 0.05) after correction for multiple testing, with positive associations in bright red and negative associations in bright blue. The standardised regression coefficients ($\hat{\beta }$) are shown with units in standard deviations for each covariate.

**Fig. 2:**
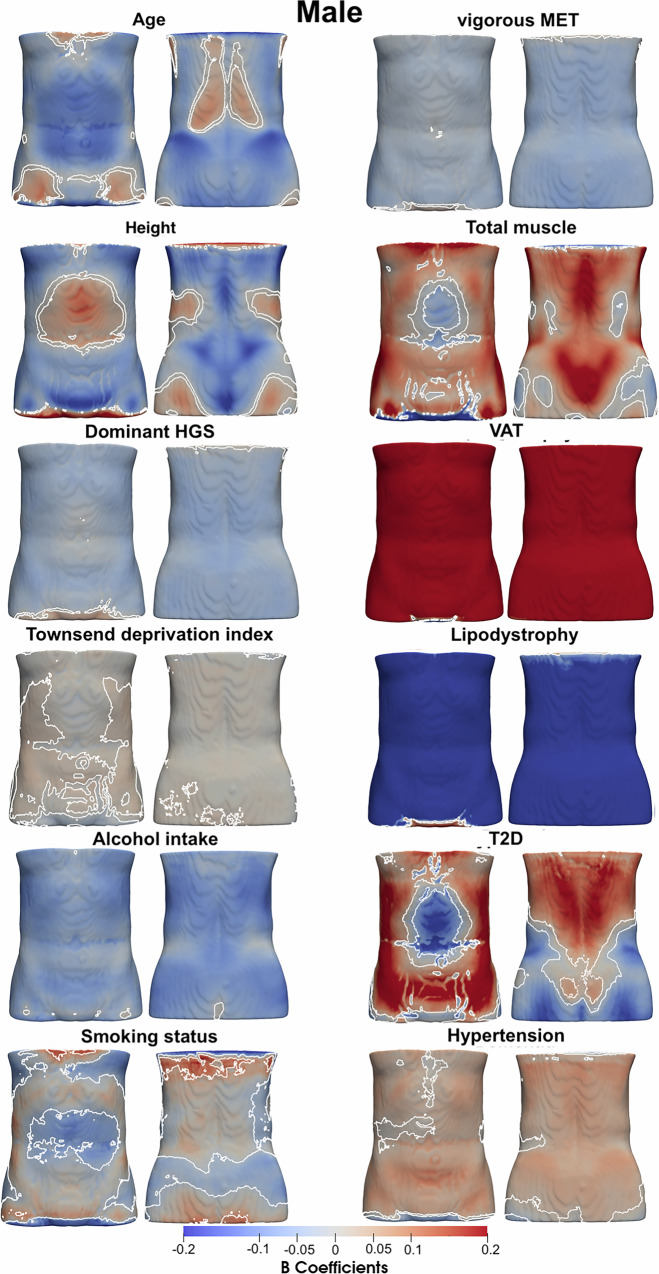
Three-dimensional statistical parametric maps (SPMs) of ASAT morphology, projections are anterior (left plots) and posterior views (right plots). The SPMs show the local strength of association for each covariate in the model with ASAT thickness for male participants (*N* = 18,470). White contour lines indicate the boundary between statistically significant regions (*p* < 0.05) after correction for multiple testing, with positive associations in bright red and negative associations in bright blue. The standardised regression coefficients ($\hat{\beta }$) are shown with units in standard deviations for each covariate.

Our spatial analysis revealed that associations between ASAT thickness and anthropometric or health-related variables are region-specific, rather than uniformly distributed across the 3D ASAT surface (Table 1, Supplementary Fig. S4 and Figs. 1 and 2). ASAT thickness was negatively associated with age, showing an overall median regression coefficient of −0.13 mm/year in women and −0.07 mm/year in men. The area where these coefficients were statistically significant covering 90.4% of the ASAT vertices in women and 84.4% in men. HGS on the dominant hand showed statistically significant negative associations with ASAT thickness, covering 88.4% and 83.6% of the ASAT vertices in the female and male participants, respectively. Lifestyle factors, including alcohol intake frequency and vigorous MET, showed statistically significant negative associations in both men and women. Smoking status was overall negatively associated in women participants covering 98% of the ASAT vertices, while in male participants, it covered 47.7%. Total muscle showed an overall positive association with ASAT thickness covering 73.7% of the ASAT vertices in women and 71.5% in men. VAT showed a median regression coefficient of 3.55 mm/l (significant area: 99.6%) with ASAT thickness in women and 1.47 mm/l (significant area: 98.5%) in men. Lipodystrophy was negatively associated with ASAT thickness covering 84.3% of ASAT in women and 87% in men.

**Table 1:** Significance areas for covariates in the MUR model for the anthropometric variables of the model for the ASAT thickness (mm) for each gender (19,418 female and 18,470 male participants).

	Female (*N* = 19,418)		Male (*N* = 18,470)	
	$\hat{\beta \,} < \,0$	$\hat{\beta }\, > \,0$	$\hat{\beta \,} < \,0$	$\hat{\beta }\, > \,0$
	Median regression coefficient (IQR, Significance area in %)			
Age (years)	−0.13 (0.12, 90.38%)	0.02 (0.03, 6.65%)	−0.07 (0.08, 84.43%)	0.026 (0.03, 10.19%)
Height (cm)	−16.67 (18.00, 77.37%)	6.76 (11.80, 18.82%)	−8.27 (8.75, 57.22%)	6.75 (14.38, 35.71%)
Dominant HGS (kg)	−0.05 (0.04, 88.42%)	0.01 (0.01, 0.53%)	−0.04 (0.02, 83.58%)	0.01 (0.01, 9.34%)
Townsend deprivation index	−0.04 (0.02, 7.47%)	0.04 (0.02, 21.76%)	−0.04 (0.02, 9.84%)	0.05 (0.03, 19.63%)
Alcohol intake frequency	−0.81 (0.56, 97.33%)	0.14 (0.02, 0.06%)	−0.58 (0.31, 98.56%)	0.13 (0.03, 0.06%)
Smoking status	−1.37 (1.03, 97.92%)	–	−0.61 (0.67, 47.73%)	0.52 (0.27, 2.56%)
Vigorous MET (hours/week)	−0.05 (0.04, 96.76%)	–	−0.03 (0.02, 83.39%)	0.01 (0.01, 4.77%)
Total muscle (l)	−0.21 (0.19, 20.52%)	0.46 (0.37, 73.72%)	−0.30 (0.42, 21.56%)	0.23 (0.25, 71.53%)
VAT (l)	−0.14 (0.17, 0.26%)	3.55 (2.05, 99.58%)	−0.24 (0.28, 1.27%)	1.47 (1.07, 98.54%)
Lipodystrophy	−1.71 (4.24, 84.27%)	0.25 (0.26, 2.19%)	−3.14 (2.34, 86.96%)	0.50 (0.74, 3.44%)
T2D	−1.43 (1.45, 64.96%)	0.69 (0.63, 21.97%)	−0.68 (0.53, 21.68%)	0.70 (0.64, 59.41%)
Hypertension	−0.06 (0.10, 0.68%)	0.44 (0.37, 84.24%)	−0.23 (0.16, 11.77%)	0.30 (0.20, 71.22%)

The total significance area has been split into areas of positive and negative associations. The regression coefficients ($\hat{\beta }$) are presented as median (interquartile range—IQR) across all vertices of the ASAT surface and the significance areas as a percentage (%) of the vertices with statistically significant associations.

A diagnosis of T2D in female participants was associated with ASAT thickness, with a median regression coefficient of −1.43 mm (significant area: 65%) in the upper abdominal areas and lower posterior areas (back and hips) as well as median regression coefficient of 0.69 mm, (significant area: 22%) in the lower anterior areas (hips) and upper posterior areas (back). In men, T2D was positively associated with ASAT thickness with a median regression coefficient of 0.70 mm, covering 59.4% of the lower anterior areas (hips and lower abdominal), upper posterior areas (upper back) and left and right posterior areas, as well as a negative association with a median regression coefficient of −0.68, covering 21.7% of the abdominal areas and lower posterior areas (hips). Hypertension was overall positively associated with ASAT thickness with a median regression coefficient of 0.44 mm (significant area: 84.2%) in women and 0.30 mm (significant area: 71.2%) in men.

### Longitudinal changes in ASAT thickness

**Fig. 3:**
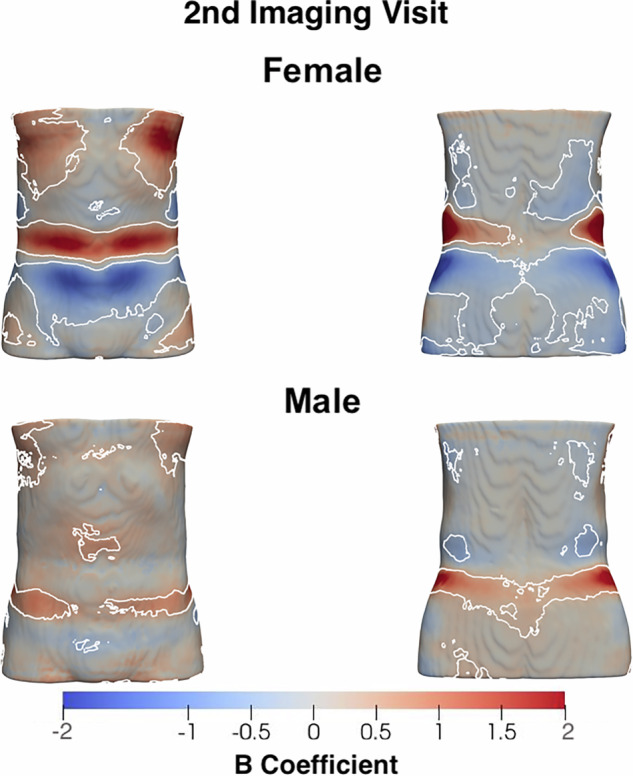
Three-dimensional statistical parametric maps (SPMs) of ASAT morphology, projections are anterior (left plots) and posterior views (right plots). The SPMs show the local strength of association of ASAT thickness and the imaging visit for the male (*N* = 1186) and female participants (*N* = 1200). White contour lines indicate the boundary between statistically significant regions (*p* < 0.05) after correction for multiple testing, with positive associations in bright red and negative associations in bright blue. The standardised regression coefficients ($\hat{\beta }$) are shown with units in standard deviations for each covariate.

Among the 2386 participants who underwent a second imaging visit (1205 women; see Supplementary Fig. S1), we observed modest median changes in ASAT thickness between visits (Supplementary Fig. S5). Linear mixed-effects models showed no significant influence of clinical conditions or diseases on longitudinal changes in ASAT thickness. Nonetheless, statistically significant regional changes were detected at follow-up (Fig. 3). For instance, women exhibited a median decrease of −0.59 mm in ASAT thickness in the lower body and upper back, alongside a median increase of 0.79 mm in the chest and waist regions. In men, ASAT thickness showed a statistically significant median increase of 0.66 mm, particularly around the lower abdomen and upper chest (Supplementary Table S3).

### Survival analysis

Here we identified 1443 participants with CVD, 926 participants with hypertension and 219 participants with T2D. We created CPH models for each disease outcome adjusted for anthropometric variables and compared the performance of two models; one including ASAT volume (the volume model); the other including the first four PC scores derived from the SPCA, accounting for over 60% of the variation in ASAT thickness in all disease outcomes and for both genders. Note that due to the small number of cases of T2D (*N* = 84) in women, CPH models were not applied in this cohort.

We show that increased VAT volume was a risk factor for CVD, hypertension and T2D (supplementary Figs. S6 and S7). We found that in men, increased ASAT volume was a risk factor for CVD (hazard ratio (HR): 1.20, 95% CI: [1.09–1.33], *p* = 0.001) and hypertension (1.19, [1.06–1.34], *p* = 0.017) (Supplementary Fig. S7). The ASAT thickness model outperformed the volume model for hypertension in women, and for hypertension and T2D in men (Supplementary Table S4).

**Fig. 4:**
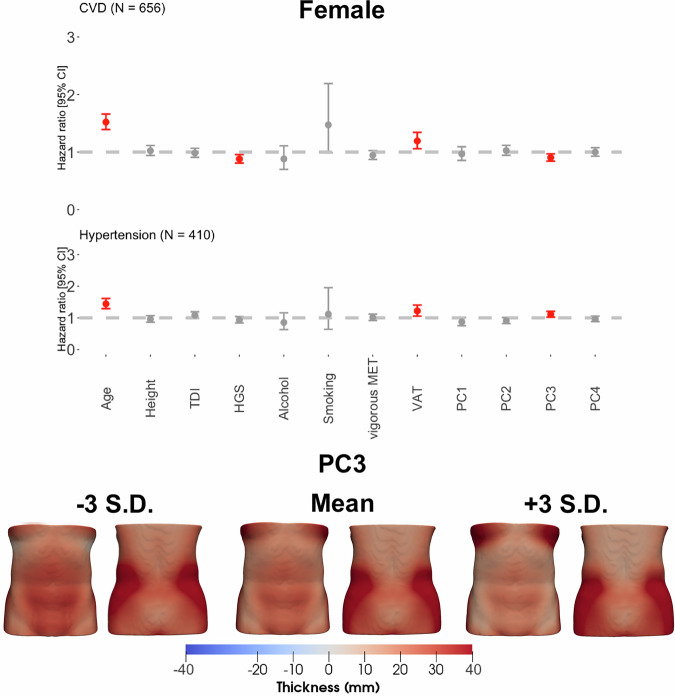
Hazard ratios and 95% CIs for CVD and hypertension outcomes for the female participants, adjusted for age, ethnicity, height, dominant HGS, Townsend deprivation index, alcohol intake frequency, smoking status, vigorous MET, VAT volume, and the first four PC scores of the ASAT thickness. Statistically significant associations (*p* < 0.05) are shown in red. The PCs are visualised below the hazard ratios, showing the minimum and maximum deviations from the average female ASAT thickness mapped onto the average female shape.

**Fig. 5:**
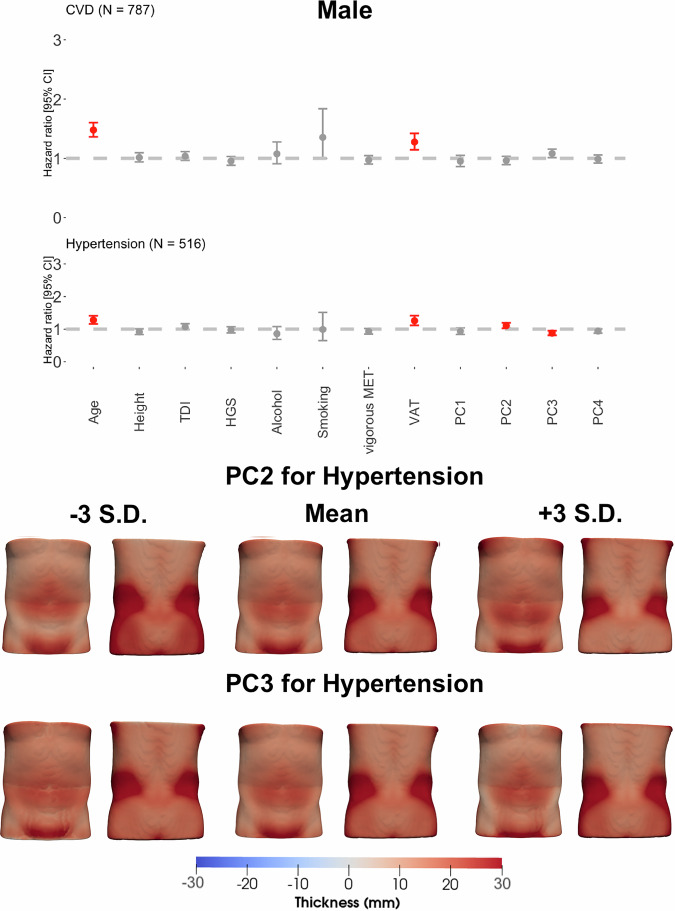
Hazard ratios and 95% CIs for CVD and hypertension outcomes for the male participants, adjusted for age, ethnicity, height, grip strength in the dominant hand, Townsend deprivation index, alcohol intake frequency, smoking status, vigorous MET, VAT volume, and the first four PC scores of the ASAT thickness. Statistically significant associations (*p* < 0.05) are shown in red. The PCs are visualised below the hazard ratios, showing the minimum and maximum deviations from the average male ASAT thickness mapped onto the average male shape.

To enhance the interpretability of the mesh-derived phenotypes, we examined the anatomical relevance of the key principal components (PCs), which likely capture distinct patterns of fat distribution linked to cardiometabolic risk. In particular, we found that in women, PC3 of ASAT thickness—reflecting variation in fat distribution between the abdomen and the chest/hips, was significantly associated with increased risk of cardiovascular disease (CVD) (HR: 0.90, 95% CI: 0.84–0.97, *p* = 0.023) and hypertension (HR: 1.11, 95% CI: 1.03–1.21, *p* = 0.045) (Fig. 4). In men PC2 score of ASAT thickness, representing the variations between the hips and the chest/lower abdomen, was positively associated with risk of hypertension (1.10, [1.02–1.19], *p* = 0.045), indicating greater ASAT thickness in the chest and lower abdomen compared to the hips, whereas PC3, representing the distribution between waist and the hips, was associated with lower risk of hypertension (0.88, [0.82–0.96], *p* = 0.014) (Fig. 5). A lower risk of T2D was associated with PC2, which in this cohort represented the distribution in ASAT thickness between the chest/lower abdomen and the hips (0.81, [0.71–0.92], *p* = 0.007) (Supplementary Fig. S8). To further assess disease-specific variations, we included the first 25 PC scores from SPCA, capturing over 80% of ASAT thickness variation in CVD and hypertension. In women, PC5 and PC11, representing abdomen-to-chest/hip variations, were risk factors for hypertension (PC5: HR = 0.86, *p* = 0.0016; PC11: HR = 1.18, *p* = 0.0013, c-index = 0.68). In men, only PC3 was associated with lower hypertension risk, aligning with the thickness model (*p* = 0.0021, c-index = 0.66) (Fig. S9). To aid interpretation, the ASAT thickness variations represented by each PC (–3 to +3 standard deviation) are visualised in Supplementary Video S1.

## Discussion

This study offers a new perspective on ASAT using advanced phenotypic and computational modelling techniques. By using ASAT segmentations of 37,968 UK Biobank participants, we mapped local variations in ASAT thickness to explore associations with anthropometric traits and disease factors. Previous research using similar SPM techniques has identified associations between phenotypic and genetic variations in specific anatomical regions [10, 11]. By extending these techniques to ASAT enhances our understanding of the complex relationships among fat distribution, anthropometric characteristics, and health conditions.

Generally, ASAT thickness is measured using various methods, include physical measurements using callipers [26], direct measurement using a measuring scale during abdominal surgery [27] or at post-mortem [28], or by ultrasound [29]. Studies using these approaches have suggested that increased ASAT thickness and fatty liver are associated with NASH [28], can predict metabolic disease and gestational diabetes [30] or predict infection in incisional surgical sites [27]. However, weight loss studies suggest changes in the ASAT thickness may not reflect those measured by a more comprehensive assessment of volume, mass or area [31]. Furthermore, little attention has been paid to specific regional variations in ASAT thickness, and how that may relate to disease. The SPM method implemented in this study reveals significant regional ASAT variations, offering a more detailed view of how ASAT affects health.

We demonstrated statistically significant negative associations between age and ASAT thickness in both men and women. Previous studies investigating age-related changes in regional fat distribution have shown that, despite an increase in visceral adiposity, subcutaneous AT tends to decrease as individuals age as the ability to store lipids subcutaneously diminishes [32]. Indeed, we found a negative association between ASAT thickness and the presence of lipodystrophy which supports this rationale [33]. We also found distinct sex-specific associations with VAT, underscoring differences in its impact on ASAT thickness. Specifically, in men, each unit increase in litres of VAT volume was associated with a median increase of 1.5 mm in ASAT thickness, whereas in women, the corresponding increase was substantially higher, with a median of 3.5 mm in ASAT thickness per litre increase in VAT volume. Sex-specific differences in fat distribution diminish after menopause, often leading to an increased risk of metabolic disease [34, 35]. Further work may be needed to determine how these changes relate to menopause and the risk of metabolic disease.

We found statistically significant negative associations between dominant HGS and ASAT thickness, something that has previously been observed in forearm subcutaneous AT [29]. Similarly, we found vigorous physical activity to be associated with lower ASAT thickness. However, we found that total muscle volume was significantly associated with increased ASAT thickness in both sexes. This apparent paradox between strength and fitness being associated with reduced ASAT, and increased muscle volume associated with increased ASAT has previously been reported [36]. Obesity may be accompanied by increased skeletal muscle mass [36] in older age, resulting in impaired physical function [37].

Alcohol intake frequency was associated with lower ASAT thickness in both men and women. Previous CT studies showed that women consuming moderate alcohol had lower subcutaneous fat [38]. Smoking was also associated with lower ASAT thickness in women, where the effect was smaller in men. This aligns with previous observations showing a negative relationship between smoking and abdominal obesity [39, 40].

Unlike most previous studies of ASAT thickness, the methods employed here enable us to look at how it varies regionally. For instance, while T2D was associated with reduced ASAT thickness in women across most anatomical regions, in men the majority of the associations showed increased thickness predominantly in the hips, lower abdominal, and upper back. Previous studies have reported that while accumulation of VAT is linked to higher metabolic risk and overall mortality, expansion of subcutaneous AT improves insulin sensitivity and reduces the risk of T2D [41, 42]. Both male and female participants with hypertension showed a positive association with ASAT thickness in most anatomical regions in the abdomen. Previous studies have associated elevated subcutaneous fat to hypertension, although VAT is more strongly related to metabolic risk in most populations [43]. However, in African American men both ASAT and VAT contribute to the development of hypertension to a similar degree [44], suggesting a more important role for ASAT in this population.

We examined whether preexisting chronic diseases influenced changes in ASAT thickness over ~2.5 years. No clear disease-related effects were observed, likely due to the relatively healthy baseline status of participants and limited disease progression during follow-up. However, in women, we noted a decrease in ASAT thickness in the lower posterior, anterior regions, and upper back, along with an increase in the upper posterior, suggesting a shift toward more centralised fat distribution with age.

While limited by short follow-up and absence of hormonal data, prior research indicates that age-related hormonal changes, particularly around menopause, as well as lifestyle factors like physical activity and alcohol intake, may drive ASAT redistribution. One longitudinal study reported that postmenopausal women aged 50–69 gained subcutaneous fat, while those over 70 lost it over 6 years [45]. This likely reflects hormonal shifts during menopause, where falling oestrogen and rising gonadotropins promote fat accumulation, whereas stabilised hormone levels in older women may lead to reduced fat gain [46]. Longer-term studies are needed to better understand these dynamics and the role of chronic disease in ASAT change.

We investigated the risk of incident disease adjusted for relevant anthropometric variables, comparing ASAT volume with ASAT thickness. We demonstrated that the ASAT thickness model outperformed the ASAT volume model for CVD and hypertension in women and for hypertension and T2D in men. We showed that, unlike conventional measures, ASAT thickness revealed more localised fat variations. In men, ASAT thickness variations corresponding to the variations in the bulkiness of the chest and lower abdomen, were positively associated with the risk of hypertension, whereas greater distribution across the hips compared to the waist was associated with a lower risk of hypertension. This reflects previous work, which identified waist-to-hip ratios as critical markers of cardiovascular risk [47, 48]. In women, ASAT thickness variations representing the accumulation of abdominal thickness versus the chest and hips were also significantly associated with risk of cardiometabolic disease, which highlights the importance of fat distribution in metabolic disorders [49]. A higher hip-to-abdominal fat ratio suggests a more favourable fat distribution, potentially reducing cardiometabolic risk factors [50]. Additionally, smaller waist and hip sizes are often linked to lower CVD mortality risk [51] with stronger associations reported in women than in men [52].

This study has several limitations. Although the UK Biobank offers a large sample, it is subject to selection bias, with participants generally healthier than the broader UK population and predominantly of European ancestry. This, along with the exclusion of younger individuals and more severe cases, may limit the generalisability of our findings [53, 54]. While higher adipose tissue (AT) in women has been linked to reduced dementia risk [55], the low prevalence of such conditions in the cohort limited our ability to detect associations between ASAT thickness and brain health. Future studies should explore these relationships in more detail. Additionally, we excluded ~6,000 participants due to missing anthropometric or lifestyle data, opting not to use multiple imputation in order to minimise model-based bias. The incidence of new type 2 diabetes (T2D) cases during follow-up was low (~0.4%), reducing statistical power for detecting significant associations. Finally, the follow-up period for health outcomes was limited to 4.6 years post-imaging, which may constrain the power of time-to-event analyses.

## Conclusions

Our findings support the growing body of literature highlighting the scientific and clinical value of shape analysis in assessing abdominal subcutaneous adipose tissue (ASAT). We identified significant associations between ASAT thickness and disease outcomes, including type 2 diabetes (T2D) and hypertension. Additionally, shape features derived from 3D ASAT morphology were predictive of future disease events, underscoring their prognostic potential. These results demonstrate the utility of morphometric approaches for improving our understanding of fat distribution and its role in disease risk. Such methods may enhance future population-based studies by uncovering links between physiological, genetic, and environmental factors influencing adipose tissue structure and function.

## Supplementary information

Supplementary_Material

Video S1

## Data Availability

Our research was conducted using UK Biobank data. Under the standard UK Biobank data sharing agreement, we (and other researchers) cannot directly share raw data obtained or derived from the UK Biobank. However, under this agreement, all of the data generated and methodologies used in this paper are returned by us to the UK Biobank, where they will be fully available. Access can be obtained directly from the UK Biobank to all bona fide researchers upon submitting a health-related research proposal to the UK Biobank (https://www.ukbiobank.ac.uk).

## Declarations

Fully anonymised images and participant metadata were obtained through UK Biobank Access Application number 44584. The UK Biobank has approval from the North West Multi-Centre Research Ethics Committee (REC reference: 11/NW/0382), and obtained written informed consent from all participants before the study. All methods were performed in accordance with the relevant guidelines and regulations as presented by the appropriate authorities, including the Declaration of Helsinki.
