# Supplementary material for: Changes in abdominal subcutaneous adipose tissue thickness associate with disease and anthropometric factors
Source: Int J Obes (Lond). 2025 Jul 9;49(9):1810–9. doi: 10.1038/s41366-025-01829-y (PMC12463666; doi:10.1038/s41366-025-01829-y)
Supplement: Supplementary file 1 — Supplementary_Material [file 41366_2025_1829_MOESM1_ESM.docx]

# **Supplementary Material**

*Data*

Participant data from the UK Biobank cohort was obtained through UK Biobank Access Application number 44584. The UK Biobank has approval from the North West Multi-Centre Research Ethics Committee (REC reference: 11/NW/0382). All methods were performed in accordance with the relevant guidelines and regulations, and informed consent was obtained from all participants. Researchers may apply to use the UKBB data resource by submitting a health-related research proposal that is in the public interest. Additional information may be found on the UK Biobank researchers and resource catalogue pages ([www.ukbiobank.ac.uk](http://www.ukbiobank.ac.uk)).

*Phenotype Definitions*

Anthropometric measurements including age, body mass index (BMI), hand grip strength (HGS), and systolic and diastolic blood pressure were taken at the UK Biobank imaging visit. Ethnicity was defined based on the self-reported ethnic background at the initial assessment visit. For the purpose of our analysis we categorised ethnic background as follows: 0 for ”White” and 1 for any other ethnic background (due to small numbers of non-white participants in this dataset [3.1%]). Sex was self-reported and included those recorded by the NHS and those obtained at the initial assessment visit. The Townsend deprivation index was calculated immediately prior to participants joining the UK Biobank.

Vigorous metabolic equivalents of task (MET) in minutes per week was taken at the UK Biobank imaging visit applying the quality control criteria described previously[^1^](https://paperpile.com/c/4GPqFd/aNVx8). Questionnaire information from the UK Biobank imaging visit was used to determine alcohol intake frequency and smoking status. For our analysis, we categorised alcohol intake frequency as 1 for “Daily or almost daily” and 0 otherwise and smoking status as 1 for ”Current” and 0 for “Previous” and “Never”. Fat mass ratio (FMR) was computed by dividing trunk fat % by leg fat % from dual-energy X-ray absorptiometry and a cutoff for FMR >1.2 in female and >1.7 in male participants were used to define partial lipodystrophy, as previously described[^2^](https://paperpile.com/c/4GPqFd/ZwiV). In our analysis partial lipodystrophy will be referred to as lipodystrophy.

*Disease Definitions*

Disease categories of interest, including T2D, CVD and hypertension, were selected based on their frequency within the UK Biobank. They were defined as being present if the date of diagnosis occurred at the time or before the imaging visit. A summary of the codes corresponding to the considered disease traits are provided in supplementary Table S1.

The codes for T2D were selected based on the ICD10 and self-reported codes for type-2 diabetes [^3^](https://paperpile.com/c/4GPqFd/AfLA). Codes for CVD were selected based on the general presence of cardiac disease as previously described[^4^](https://paperpile.com/c/4GPqFd/oWcN). Hypertension was defined as self-reported of hypertensive medication use, or a prior diagnosis of hypertension, or mean blood pressure ≥ 140/90 mmHg[^5^](https://paperpile.com/c/4GPqFd/X3zIb). The codes for hypertension diagnosis were selected based on the ICD10 and the self-reported codes for hypertension. Hypertensive medication codes were selected from the medication fields, reporting as regularly taking blood pressure medication[^3^](https://paperpile.com/c/4GPqFd/AfLA).

T2D, CVD and hypertension diagnosed or reported after the imaging visit was defined based on ICD10 codes from the various sources, the date of first diagnosis made available as “first occurrence” data (Category 1712) and based on a combination of hospital records, primary care records, self-reported, and death records and the self-reported codes described in supplementary Table S1, defined after the imaging visit.

*Mass Univariate Regression Analysis*

We performed mass univariate regression (MUR) analysis using a refined version of the R package *mutools3D*[*^6^*](https://paperpile.com/c/4GPqFd/gfHlZ). To account for multiple comparisons, we applied the false discovery rate (FDR) procedure adjusted for multiple comparisons by applying the FDR procedure using the Benjamini-Hochberg method[^7^](https://paperpile.com/c/4GPqFd/DAO1K) to all the TFCE-derived p-values for each vertex and each model. The estimated regression coefficients $\hat{\beta}$ for each of the relevant covariates and their related TFCE-derived p-values, after correction for multiple testing, were then displayed at each vertex in the mesh on the whole 3D ASAT anatomy, providing the spatially-distributed associations, to adjust the p-values derived from TFCE across all vertices, using 100 permutations. The estimated regression coefficients for the relevant covariates and their corresponding TFCE-derived p-values, after correcting for multiple testing, were then spatially mapped at each vertex within the 3D ASAT anatomy mesh. This procedure allowed us to uncover associations distributed across the anatomical structure. A 3D mesh construction and phenotype mapping of 3D ASAT thickness is outlined in supplementary Figures S1 and S3.

*Statistical Shape Analysis*

Statistical shape analysis (SSA) is a technique that widely uses principal component analysis (PCA) to define the variation of the size and shape (represented using a surface mesh) across participants. Here, PCA was used to construct a low-dimensional subspace of the ASAT model representing the shape variations. Due to the large number of vertices on the 3D ASAT model, for this analysis we reduced the number of vertices to 20,000 (using VTK’s decimation algorithm[^8^](https://paperpile.com/c/4GPqFd/2uYMO)) to downsample the input mesh while preserving the topology of the original mesh. Due to having a large number of ASAT thickness values with smaller population groups, we calculated the sparse principal component analysis (SPCA) using the R package *sparsepca*[*^9,10^*](https://paperpile.com/c/4GPqFd/PZJ4d+BGLo) and extracted PC scores to characterise variations in ASAT thickness across male and female participants, separately. The first four modes of ASAT thickness variation from the PCA are presented as -3 SD, mean and +3 SD (supplementary video S1).

Supplementary Tables

| **Trait** | **ICD-10** | **Self-Reported Code (20002)** | **UK Biobank Field** |
| --- | --- | --- | --- |
| **T2D** | E11 | 1220, 1223 | - |
| **CVD** | I00-I25, I30-I52 | - | - |
| **Hypertension** | I10-I13, I15, O10 | 1065, 1072 | 6153, 6177 |

Table S1. Summary of the codes used to define disease.

|  | **Full Cohort**  **(N = 37,888)** | **Female**  **(N = 19,418)** | **Male**  **(N = 18,470)** |
| --- | --- | --- | --- |
| **Caucasian** $\boldsymbol{(n)}$ | 36,790 | 18,889 | 17,901 |
| **Age** $\boldsymbol{(yrs.)}$ | 64.03 ± 7.72  (44, 82) | 63.35 ± 7.56  (45, 82) | 64.76 ± 7.82  (44, 82) |
| **Weight** $\boldsymbol{(kg)}$ | 76.10 ± 15.03  (33, 169.7) | 68.91 ± 12.94  (33, 169.7) | 83.66 ± 13.28  (46.5, 169.2) |
| **Height** $\boldsymbol{(m)}$ | 1.69 ± 0.09  (1.35, 2.02) | 1.63 ± 0.06  (1.35, 1.95) | 1.76 ± 0.07  (1.50, 2.02) |
| **BMI** $\boldsymbol{(kg/m}^{\boldsymbol{2}}\boldsymbol{)}$ | 26.49 ± 4.33  (13.39, 62.04) | 26.03 ± 4.69  (13.39, 62.04) | 26.98 ± 3.87  (16.38, 52.67) |
| **Waist circumference** $(cm)$ | 88.20 ± 12.57  (53, 152) | 82.53 ± 11.64  (53, 144) | 94.17 ± 10.58  (63, 152) |
| **Hip circumference** $(cm)$ | 100.70 ± 8.62  (68, 176) | 100.73 ± 9.71  (68, 157) | 100.66 ± 7.30  (76.2, 176) |
| **WHR** | 0.87 ± 0.09  (0.53, 1.31) | 0.82 ± 0.07  (0.60, 1.22) | 0.93 ± 0.06  (0.53, 1.31) |
| **SBP** $\boldsymbol{(mmHg)}$ | 139 ± 18.66  (76.5, 240.5) | 136.00 ± 19.30  (77.5, 237.5) | 142.08 ± 17.42  (76.5, 240.5) |
| **DBP** $\boldsymbol{(mmHg)}$ | 78.77 ± 10.05  (37.5, 129) | 77.05 ± 9.95  (37.5, 121.5) | 80.57 ± 9.84  (46.5, 129) |
| **Dominant HGS** $\boldsymbol{(kg)}$ | 31.2 ± 10.50  (8, 90) | 23.99 ± 5.97  (8, 65) | 38.78 ± 8.75  (8, 90) |
| **Townsend deprivation index** | -1.94 ± 2.70  (-6.26, 10.10) | -1.89 ± 2.70  (-6.26, 10.10) | -1.99 ± 2.69  (-6.26, 9.74) |
| **Alcohol intake frequency** $\boldsymbol{(n)}$ | 6,479 | 2,639 | 3,840 |
| **Smoker** $\boldsymbol{(n)}$ | 1,312 | 561 | 749 |
| **Vigorous MET** $\boldsymbol{(hours/week)}$ | 6.57 ± 8.03  (0, 21) | 5.81 ± 7.68  (0, 21) | 7.36 ± 8.31  (0, 21) |
| **VAT volume** $\boldsymbol{(l)}$ | 3.95 ± 2.31  (0, 16.36) | 2.81 ± 1.55  (0, 11.49) | 5.16 ± 2.35  (0, 16.36) |
| **ASAT volume** $\boldsymbol{(l)}$ | 8.46 ± 4.63  (0.07, 38.21) | 9.82 ± 4.34  (0.07, 38.21) | 7.02 ± 3.27  (0.07, 32.58) |
| **Total muscle volume** $\boldsymbol{(l)}$ | 17.87 ± 4.63  (5.62, 36.11) | 14.07 ± 1.95  (5.62, 27.14) | 21.85 ± 3  (10.82, 36.11) |

Table S2. Summary statistics (mean ± standard deviation, minimum and maximum values) for continuous variables and counts for discrete variables in the full cohort (N=36,742). BMI: Body mass index; WHR: waist-to-hip ratio; HGS: Hand grip strength; SBP: Systolic blood pressure; DBP: Diastolic blood pressure; MET: Metabolic equivalents of task; VAT: Visceral adipose tissue; ASAT: Abdominal subcutaneous adipose tissue.

|  | **Female** (N=1,200) | | **Male** (N=1,186) | |
| --- | --- | --- | --- | --- |
|  | $\hat{\beta}<0$ | $\hat{\beta}\boldsymbol{>0}$ | $\hat{\beta}<0$ | $\hat{\beta}\boldsymbol{>0}$ |
|  | Median beta coefficients (IQR, Significance area (%)) | | | |
| **Intercept** | - | 24.76 (14.75, 99.99%) | - | 18.18 (10.42, 99.71%) |
| **Age at baseline** | -1.05 (0.75, 74.4%) | 0.32 (0.40, 3.43%) | -0.72 (0.73, 70.06%) | 0.43 (0.23, 1.77%) |
| **Height** | -1.01 (0.78, 57.81%) | 0.80 (0.87, 9.83%) | -0.78 (0.50, 42.19%) | 0.85 (0.91, 23.45%) |
| **Dominant HGS** | -0.38 (0.15, 10.98%) | - | -0.44 (0.17, 8.8%) | 0.21 (0.29, 0.13%) |
| **Townsend deprivation index** | - | - | - | - |
| **Alcohol intake frequency** | - | - | -1.28 (0.44, 3.18%) | - |
| **Smoking status** | - | - | -6.08 (0.30, 0.02%) | 2.56 (0.87, 0.08%) |
| **Vigorous MET** | -0.33 (0.18, 32.34%) | - | -0.34 (0.17, 14.6%) | 0.17 (0.14, 0.05%) |
| **Total muscle** | -0.73 (0.49, 10.63%) | 0.75 (0.35, 39.86%) | -1.28 (1.04, 17%) | 0.84 (0.67, 41.76%) |
| **VAT** | -0.39 (0.28, 0.14%) | 5.42 (3.13, 98.74%) | -0.68 (0.49, 0.92%) | 3.70 (2.51, 98.17%) |
| **Lipodystrophy** | -4.62 (4.78, 48.06%) | 1.70 (0.71, 0.45%) | -3.04 (2.26, 70.55%) | 0.75 (0.73, 0.3%) |
| **T2D** | -4.02 (0.43, 0.23%) | 1.48 (0.86, 0.5%) | -3.30 (1.77, 2.4%) | 1.34 (1.22, 0.17%) |
| **Hypertension** | - | - | - | - |
| **Second Imaging Visit** | -0.59 (0.53, 22.47%) | 0.79 (0.77, 21.21%) | -0.53 (0.27, 1.8%) | 0.66 (0.38, 13.63%) |
| **Lipodystrophy * 2nd Visit** | -2.14 (0.12, 0%) | NA (NA, 0%) | -3.03 (1.54, 0.03%) | 2.44 (0.75, 0.09%) |
| **T2D * 2nd Visit** | -1.31 (0.55, 0.09%) | 2.14 (1.06, 0.16%) | -2.24 (1.21, 0.2%) | 2.17 (0.04, 0.01%) |
| **Hypertension * 2nd Visit** | - | - | - | - |

Table S3. Significance areas for covariates in mixed-effect models for the anthropometric variables of the model for the ASAT thickness (mm) by gender (1,200 female and 1,186 male participants). The total area has been split into areas of positive and negative associations. The regression coefficients ($\hat{\beta}$) are presented as median (interquartile range - IQR) across all vertices of the ASAT surface and the significance areas as a percentage (%) of the vertices with statistically significant associations. All continuous variables used as fixed effects were standardised before analysis.

|  | **Models** | | | |
| --- | --- | --- | --- | --- |
| **Disease Cohort (N)** | **ASAT Volume** | | **ASAT Thickness** | |
|  | C-index | AIC | C-index | AIC |
| **Female** | | | | |
| **CVD (656)** | 0.66 | 12016.5 | 0.66 | 12014.4 |
| **Hypertension (410)** | 0.65 | 7528.68 | 0.66 | 7521.9 |
| **Male** | | | | |
| **CVD** | 0.65 | 14009.48 | 0.65 | 14021.26 |
| **Hypertension (516)** | 0.64 | 9182.83 | 0.65 | 9180.36 |
| **T2D (135)** | 0.75 | 2493.32 | 0.76 | 2485.44 |

Table S4. Summary of the concordance index (C-index) and the Akaike’s Information Criterion (AIC) from the Cox proportional hazard models for all disease groups (CVD, hypertension and T2D) separated by gender. Disease cohort with small numbers of cases were not analysed.

Supplementary Figures


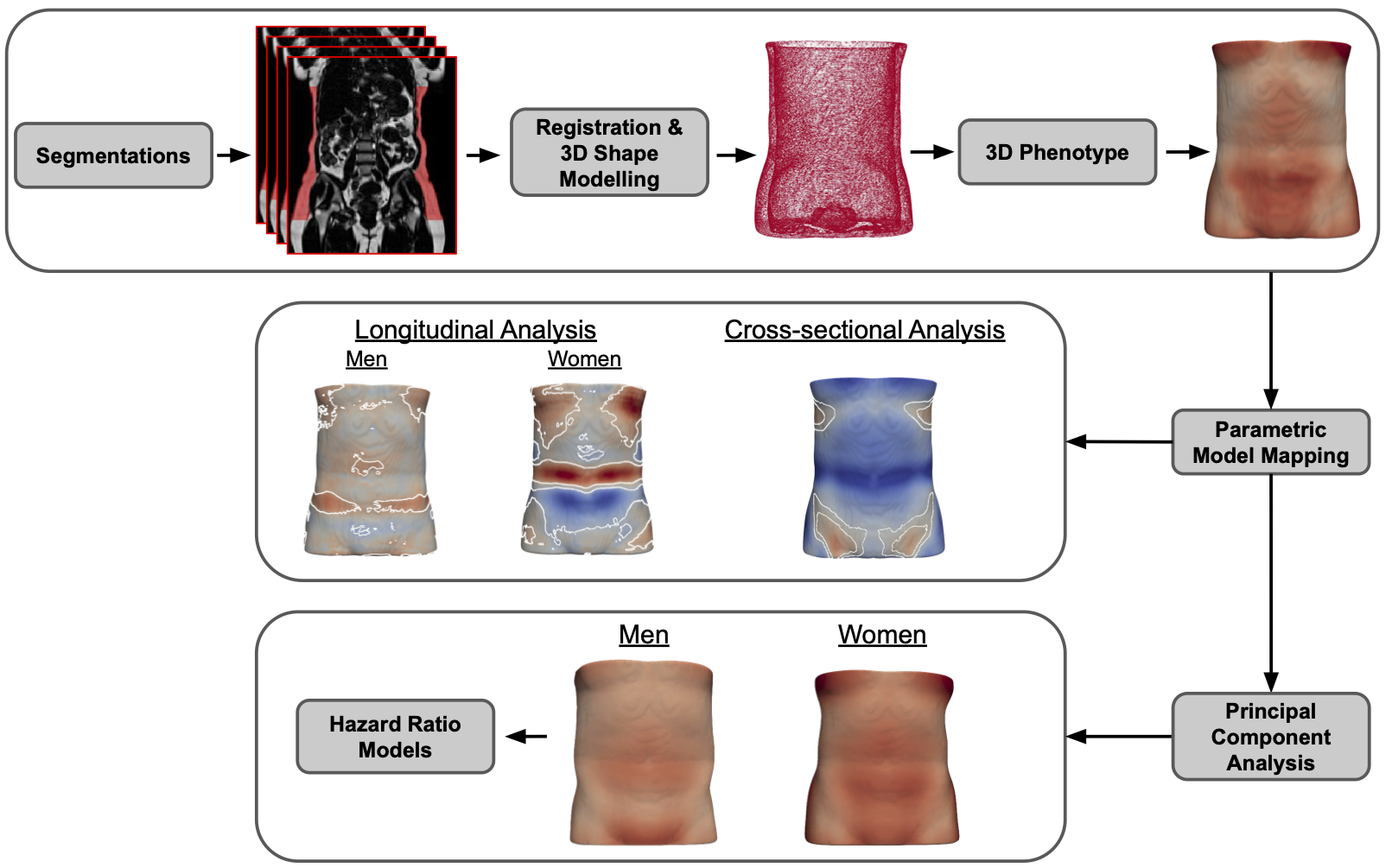


Figure S1. Overview 3D mesh construction, registration, phenotype mapping and analysis workflow. Dixon MRI images from UK Biobank are used to segment ASAT, register images to a common space, and create a 3D reference ASAT template mesh. 3D ASAT thickness is computed, mapped to individual meshes, and analysed via mas univariate regression models cross-sectionally and longitudinally, with results visualised on the template mesh. The principal component analysis is used to reduce dimensionality of the 3D ASAT thickness, and hazard ratio models are used to assess associations between 3D phenotypes and incident disease risk for both men and women.


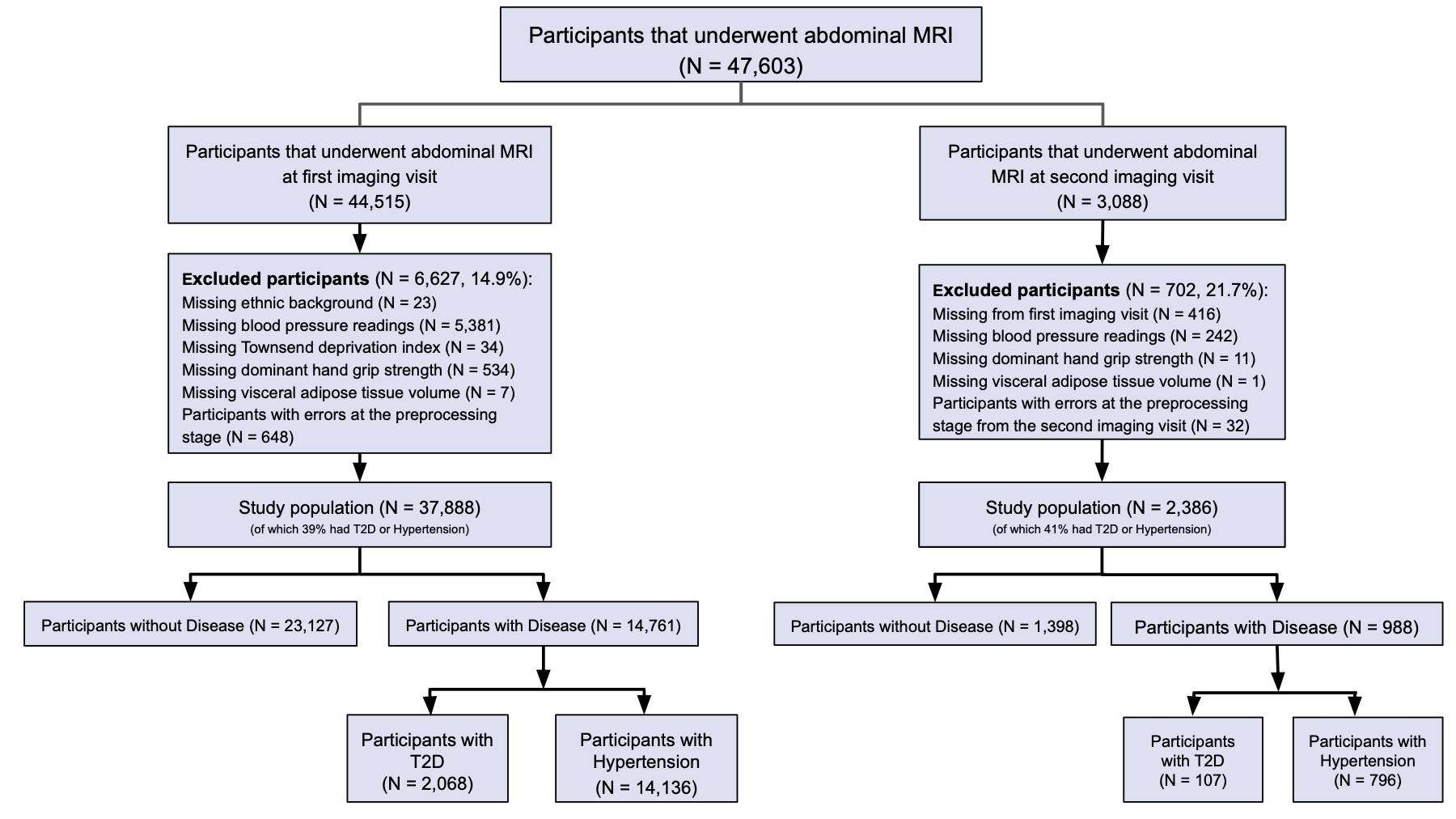


Figure S2. Flow diagram of the study population. T2D: Type-2 diabetes.


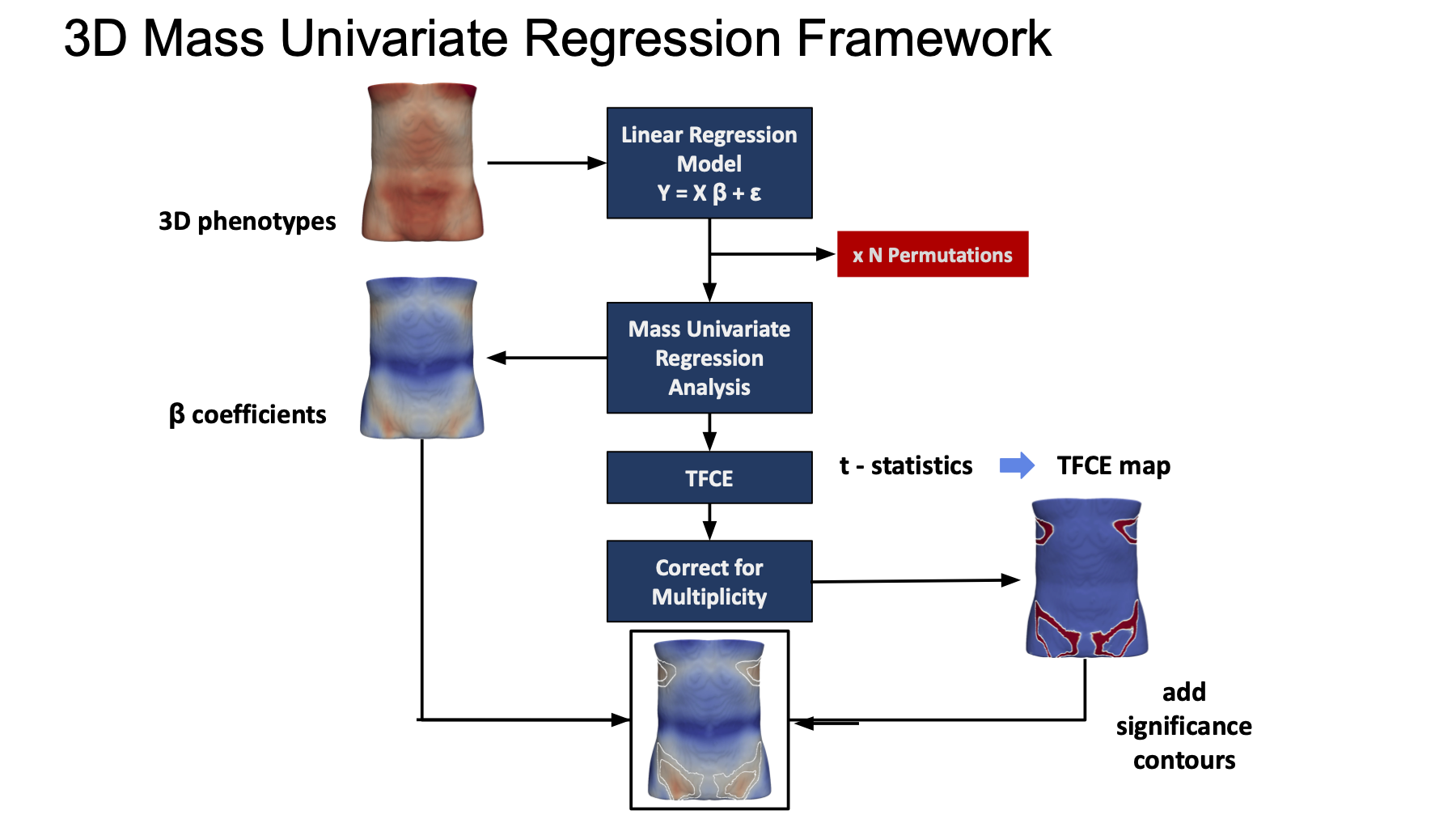


Figure S3. Flow diagram for the mass univariate regression (MUR) analysis of the 3D ASAT thickness phenotypes. The 3D phenotypes are used to construct the linear regression model. MUR analysis produces parameter estimates ($\hat{\beta}$) and their null distribution via permutation. Threshold free cluster enhancement (TFCE) is applied to the $t$-statistics from the regression analysis to produce a significance threshold. The associated TFCE-derived $p$-values are corrected for multiple comparisons and mapped onto the ASAT template mesh for visualisation. This diagram was modified from Biffi et al.[^6^](https://paperpile.com/c/4GPqFd/gfHlZ).


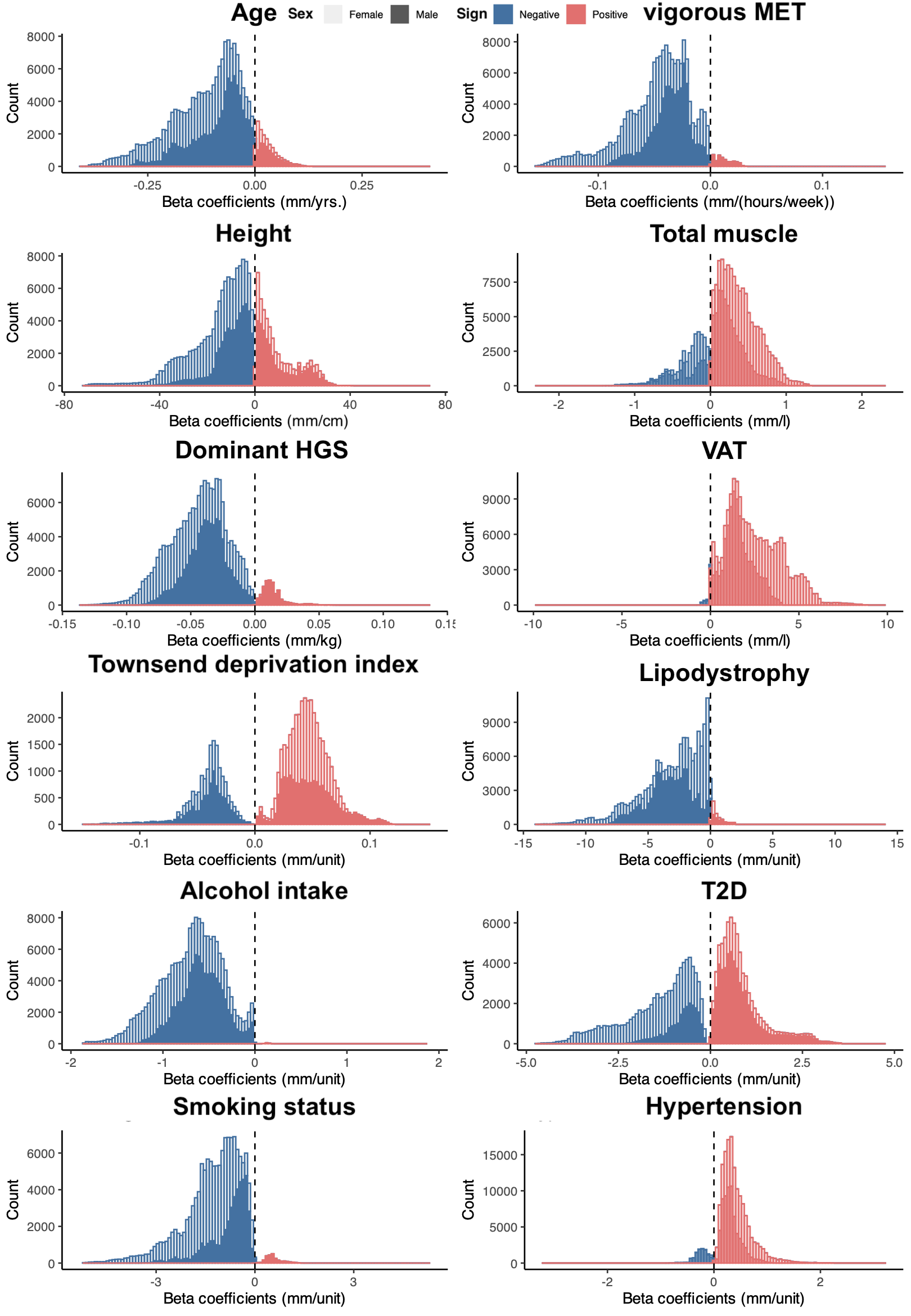


Figure S4. Histograms of the statistically significant regression coefficients across the vertices (approximately 90,000) of the ASAT for each covariate in the model on the full cohort separated by gender. Positive associations are in red and negative associations in blue, with female participants (N=19,418) in a lighter colour and male participants (N=18,470) in a darker colour. The regression (beta) coefficients are provided with units in standard deviations for each covariate.


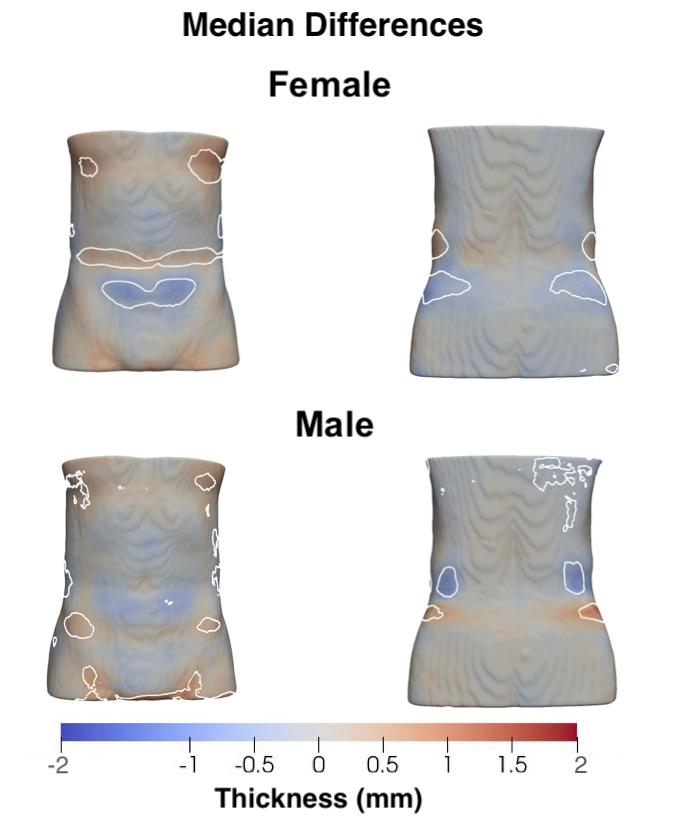


Figure S5. Three-dimensional ASAT morphology, showing the median changes in the ASAT thickness between the first and second imaging visit for the male (N=18,470) and female participants (N=19,418). Projections are anterior (left plots) and posterior views (right plots). White contour lines indicate the boundary between statistically significant regions (p < 0.05) after correction for multiple testing, with positive changes shown in bright red and negative changes shown in bright blue.


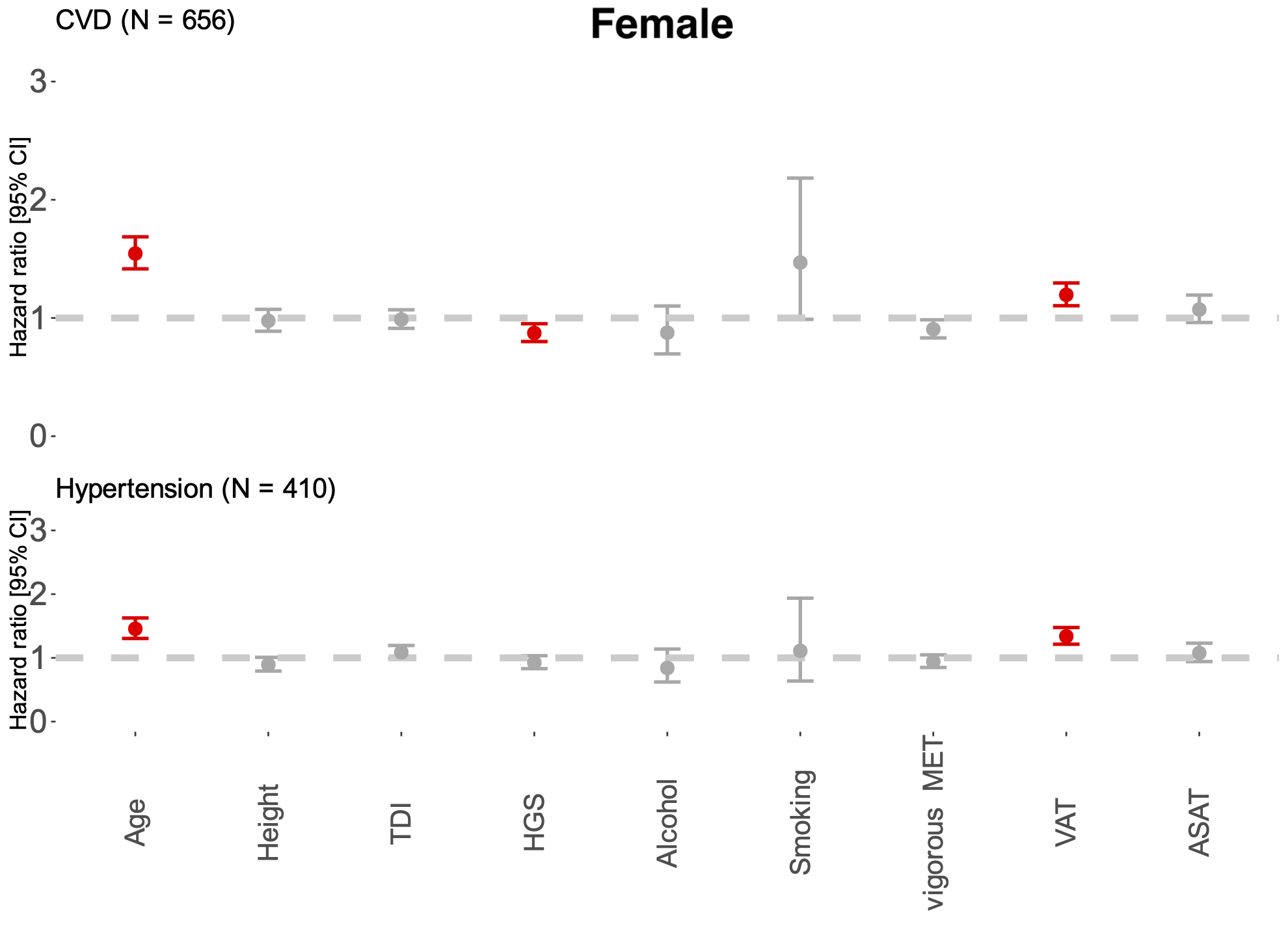


Figure S6. Hazard ratios and 95% CIs for CVD and hypertension outcomes for the female participants, adjusted for age, ethnicity, height, grip strength in the dominant hand, Townsend deprivation index (TDI), alcohol intake frequency, smoking status, vigorous MET, VAT and ASAT volume. Statistically significant associations (p < 0.05) are shown in red.


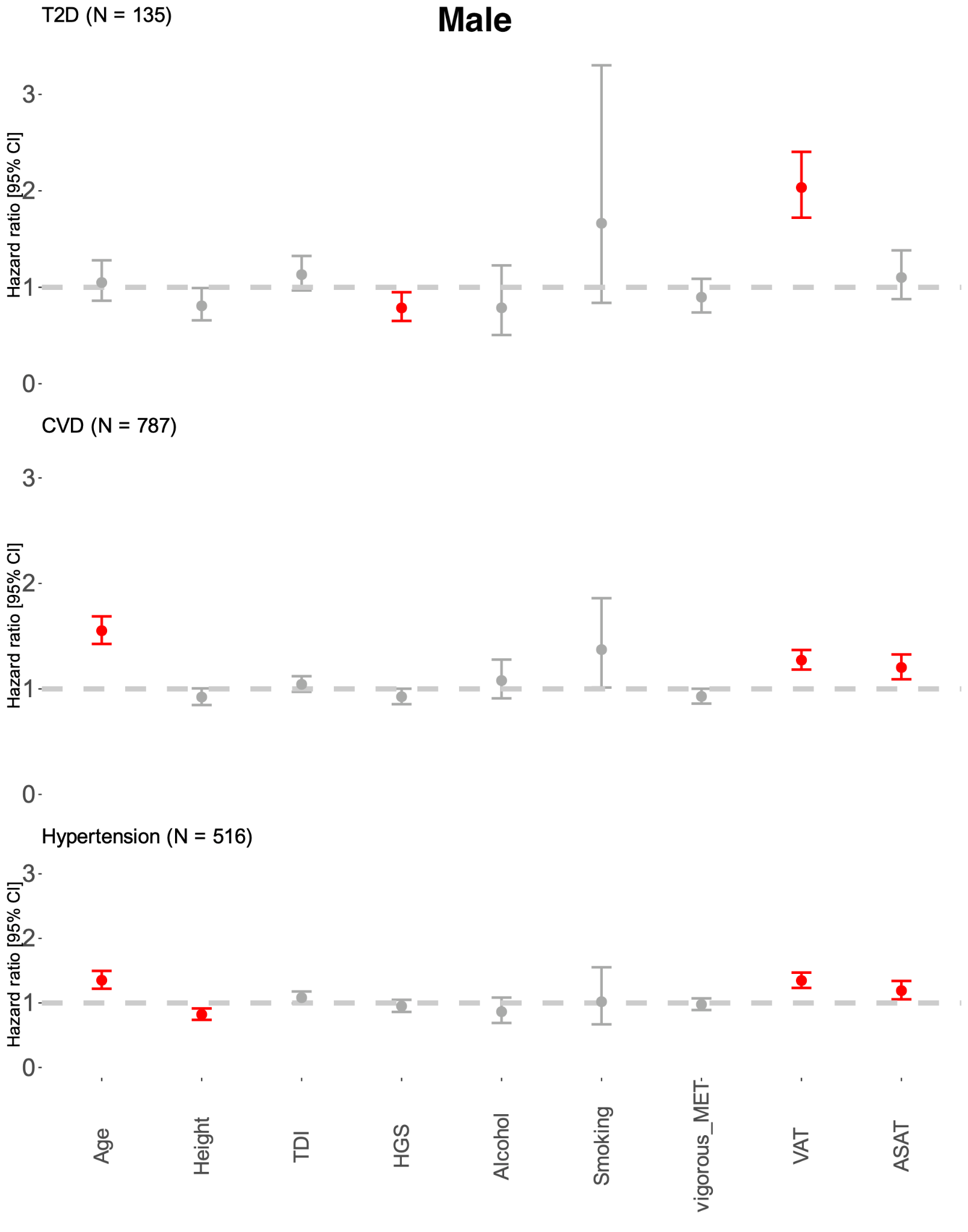


Figure S7. Hazard ratios and 95% CIs for T2D, CVD and hypertension outcomes for the male participants, adjusted for age, ethnicity, height, grip strength in the dominant hand, Townsend deprivation index (TDI), alcohol intake frequency, smoking status, vigorous MET, VAT and ASAT volume. Statistically significant associations (p < 0.05) are shown in red.


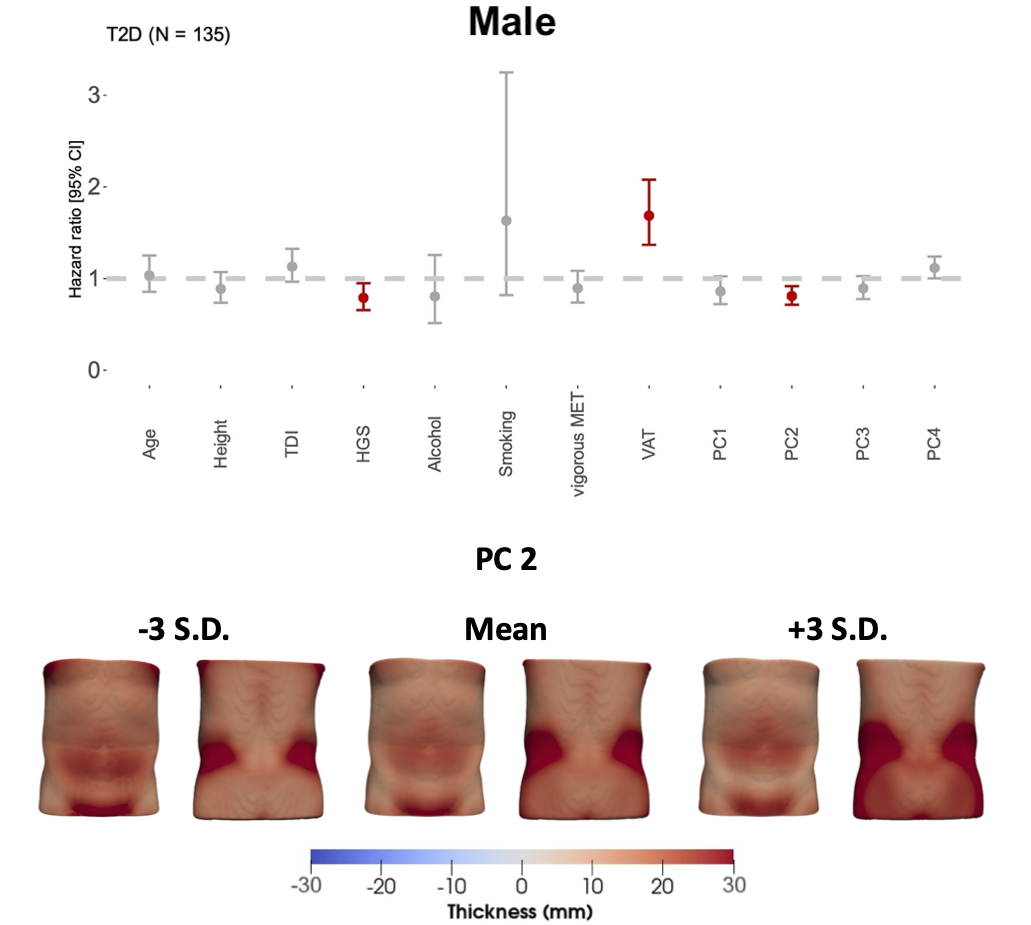


Figure S8. Hazard ratios and 95% CIs for T2D outcomes for the male participants, adjusted for age, ethnicity, height, grip strength in the dominant hand, Townsend deprivation index (TDI), alcohol intake frequency, smoking status, vigorous MET, VAT volume, and the first four PC scores for ASAT thickness. Statistically significant associations (p < 0.05) are shown in red. The PCs are visualised below the hazard ratios of the male participants, showing the minimum and maximum deviations from the average male ASAT thickness mapped onto the average male shape.


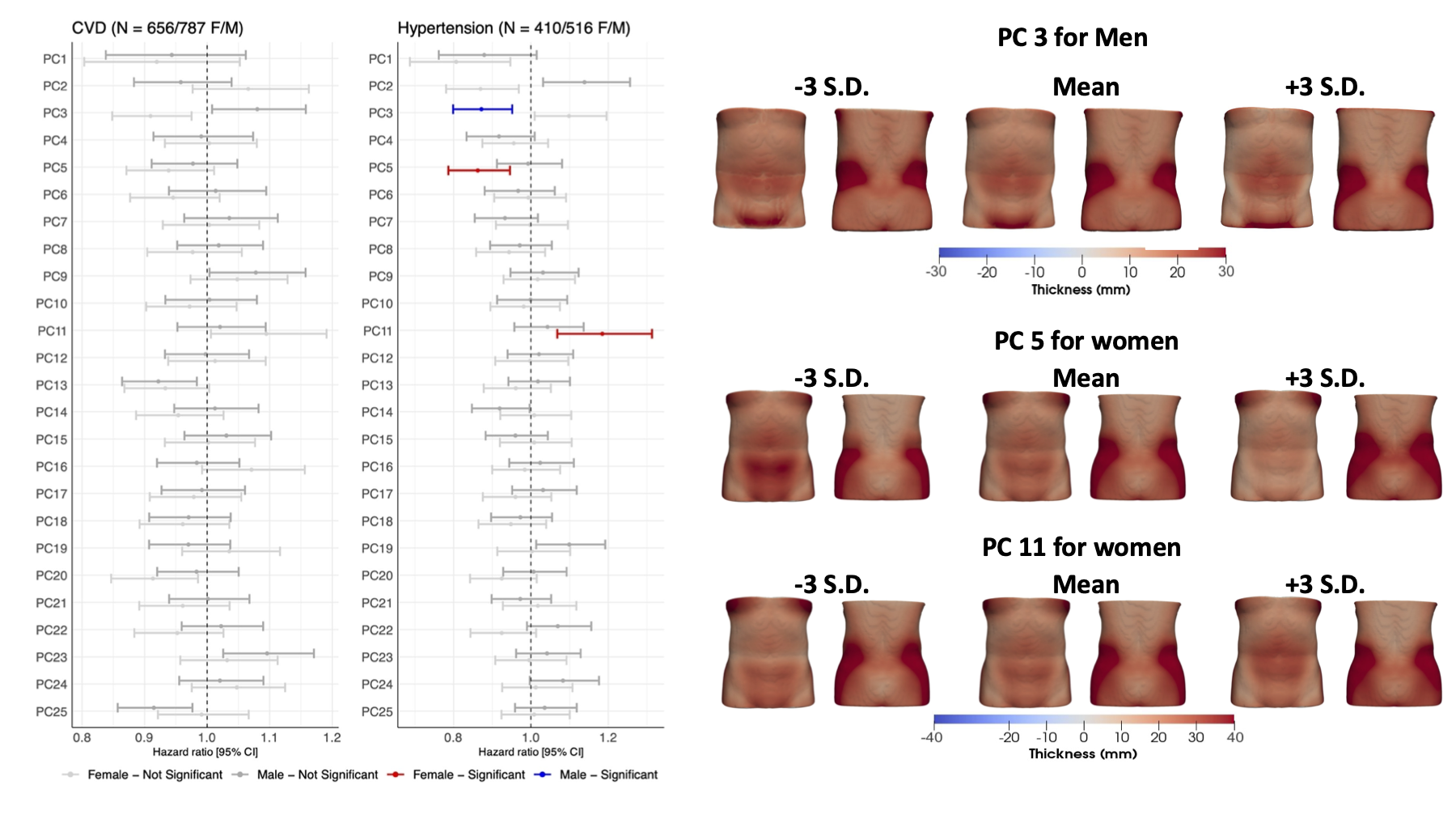
Figure S9. Hazard ratios and 95% CIs for CVD and hypertension outcomes for the male and female participants, adjusted for age, ethnicity, height, grip strength in the dominant hand, Townsend deprivation index (TDI), alcohol intake frequency, smoking status, vigorous MET, VAT volume, and the first 25 PC scores, accounting for over 80% of the variation in ASAT thickness. Statistically significant associations (p < 0.05) are shown in red for women and blue for men. The PCs are visualised below the hazard ratios of the male participants, showing the minimum and maximum deviations from the average male ASAT thickness mapped onto the average male and female shape.

Supplementary Videos


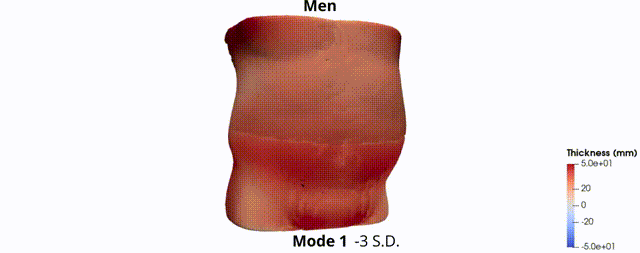

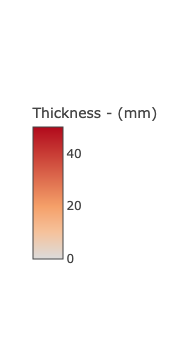


**Video S1.** The first 4 modes of shape variation for the ASAT for each gender. The mean shape (for each gender) and the shape at the +/- 3 standard deviations are displayed for each mode showing the ASAT Thickness change in mm.

**References**

1 [Thanaj M, Basty N, Whitcher B, Sorokin EP, Liu Y, Srinivasan R *et al.* Precision MRI phenotyping of muscle volume and quality at a population scale. medRxiv. 2023; : 2023.03.02.23286689.](http://paperpile.com/b/4GPqFd/aNVx8)

2 [Agrawal S, Luan J ’an, Cummings BB, Weiss EJ, Wareham NJ, Khera AV. Relationship of Fat Mass Ratio, a Biomarker for Lipodystrophy, With Cardiometabolic Traits. *Diabetes* 2024; **73**: 1099–1111.](http://paperpile.com/b/4GPqFd/ZwiV)

3 [Thanaj M, Basty N, Cule M, Sorokin EP, Whitcher B, Srinivasan R *et al.* Kidney shape statistical analysis: associations with disease and anthropometric factors. *BMC Nephrol* 2023; **24**: 362.](http://paperpile.com/b/4GPqFd/AfLA)

4 [Shah M, de A Inácio MH, Lu C, Schiratti P-R, Zheng SL, Clement A *et al.* Environmental and genetic predictors of human cardiovascular ageing. *Nat Commun* 2023; **14**: 4941.](http://paperpile.com/b/4GPqFd/oWcN)

5 [Tapela N, Collister J, Clifton L, Turnbull I, Rahimi K, Hunter DJ. Prevalence and determinants of hypertension control among almost 100 000 treated adults in the UK. *Open Heart* 2021; **8**. doi:](http://paperpile.com/b/4GPqFd/X3zIb)[10.1136/openhrt-2020-001461](http://dx.doi.org/10.1136/openhrt-2020-001461)[.](http://paperpile.com/b/4GPqFd/X3zIb)

6 [Biffi C, de Marvao A, Attard MI, Dawes TJW, Whiffin N, Bai W *et al.* Three-dimensional cardiovascular imaging-genetics: a mass univariate framework. *Bioinformatics* 2018; **34**: 97–103.](http://paperpile.com/b/4GPqFd/gfHlZ)

7 [Benjamini Y, Hochberg Y. Controlling the False Discovery Rate: A Practical and Powerful Approach to Multiple Testing. Journal of the Royal Statistical Society: Series B (Methodological). 1995; **57**: 289–300.](http://paperpile.com/b/4GPqFd/DAO1K)

8 [Schroeder W, Martin KW, Martin K, Lorensen B. *The Visualization Toolkit*. Prentice Hall, 1998.](http://paperpile.com/b/4GPqFd/2uYMO)

9 [Erichson et al. 2017.](http://paperpile.com/b/4GPqFd/PZJ4d) [Erichson BN, Zheng P, Aravkin S. Sparse principal component analysis (SPCA), https://github.com/erichson/spca, R package version 0.1.2. 2018.](about:blank)

10 [N. Benjamin Erichson, Peng Zheng, and Sasha Aravkin. sparsepca: Sparse Principal Component Analysis (SPCA). CRAN: Contributed Packages. 2018. doi:](http://paperpile.com/b/4GPqFd/BGLo)[10.32614/cran.package.sparsepca](http://dx.doi.org/10.32614/cran.package.sparsepca)[.](http://paperpile.com/b/4GPqFd/BGLo)
