# Supplementary figures and images for: Changes in abdominal subcutaneous adipose tissue thickness associate with disease and anthropometric factors
Source: Int J Obes (Lond). 2025 Jul 9;49(9):1810–9. doi: 10.1038/s41366-025-01829-y (PMC12463666; doi:10.1038/s41366-025-01829-y)

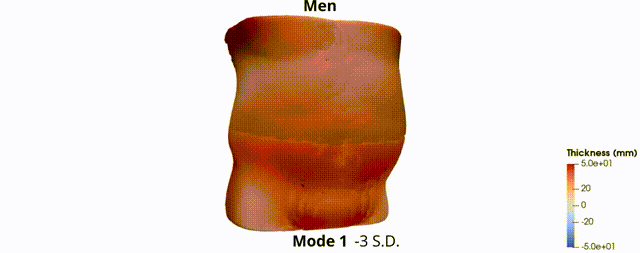

Supplement: Supplementary file 2 — Video S1 [file 41366_2025_1829_MOESM2_ESM.gif]
